# Differences in ICU Outcomes According to the Type of Anticancer Drug in Lung Cancer Patients

**DOI:** 10.3389/fmed.2022.824266

**Published:** 2022-02-14

**Authors:** Yoonki Hong, Ji Young Hong, Jinkyeong Park

**Affiliations:** ^1^Department of Internal Medicine, School of Medicine, Kangwon National University Hospital, Kangwon National University, Chuncheon, South Korea; ^2^Department of Internal Medicine, Hanlym University Chuncheon Hospital, Chuncheon, South Korea; ^3^Department of Internal Medicine, Dongguk University Ilsan Hospital, Goyang, South Korea

**Keywords:** lung cancer, mortality, intensive care unit, chemotherapy, targeted agents

## Abstract

**Purpose:**

We investigated the intensive care unit (ICU) outcomes of patients who used targeted therapy compared to those who received cytotoxic chemotherapy.

**Materials and Methods:**

This study was based on Korean administrative health insurance claims from 2015 to 2019. We extracted data on lung cancer patients (>18 years old) who were admitted to the ICU after receiving chemotherapy.

**Results:**

6,930 lung cancer patients who received chemotherapy within 30 days before ICU admission were identified; the patients received cytotoxic chemotherapy (85.4%, *n* = 5,919) and molecular targeted therapy (14.5%, *n* = 1,011). Grade 4 neutropenia was identified only in the cytotoxic chemotherapy group (0.6%). Respiratory failure requiring ventilator treatment was more common in the cytotoxic chemotherapy group than in the targeted therapy group (HR, 3.30; 95% CI, 2.99–3.63), and renal failure requiring renal replacement therapy was not significantly different between the two groups (HR, 1.57; 95% CI, 1.36–1.80). Patients who received targeted chemotherapy stayed longer in the ICU than the cytotoxic chemotherapy. The 28-day mortality was 23.4% (HR, 0.79; 95% CI, 0.67–0.90, *p* < 0.05) among patients who received targeted agents compared with 29.6% among patients who received cytotoxic chemotherapy.

**Conclusion:**

Targeted chemotherapy for lung cancer may contribute to increasing access to critical care for lung cancer patients, which may play a role in improving critical care outcomes of lung cancer patients.

## Introduction

Treatment outcomes for critically ill patients have improved, not only for patients without cancer but also for patients with cancer (1). As a result of the evolution of intensive care unit (ICU) admission strategies, the increase in adherence to sepsis bundle after sepsis survival, and efforts to apply protective lung strategies for mechanical ventilators, ICU outcomes have continuously improved over the last two decades (2). Improvements in cancer treatment outcomes caused by the advent of immunotherapies and targeted therapies allows better outcomes of ICU treatment since many cancer patients are able to recover.

Recent survival improvements with targeted agents and immunotherapies in patients with non-small cell lung cancer (NSCLC) has led physicians to more complex situations where additional factors should be considered in determining critical care for patients with lung cancer (3). The decision whether to proceed to ICU treatment including with mechanical ventilation or hemodialysis has been a complicated and difficult matter for critically ill patients with lung cancer in clinical practice. This decision may have been made reluctantly in that ICU admissions for patients with lung cancer result in high mortality rates (4), high economic burden (5, 6), and poor quality of life after ICU discharge (7). On the other hand, the decision may be positively considered in that ICU survival rates have been improved by significant advances in infection control and critical care (8). Several studies found that patients who were prescribed targeted agents were more likely to continue treatment until near death (9). Additionally, a study showed that patients who received targeted agents tended to receive more aggressive treatments near death, admission to the ICU, and mechanical ventilation (10).

Recently, several studies have showed the overall outcomes of lung cancer patients admitting to ICU according to modality receiving treatment (10, 11), helping physicians to make more careful decisions about ICU treatments for patients with lung cancer. However, little is known about the benefits of ICU treatment in patients with lung cancer who are were treated with targeted therapies or immunotherapies. Therefore, we investigated the outcomes of ICU admission among patients with lung cancer from a national population-based database according to the chemotherapeutic treatment.

## Materials and Methods

### Data Source

This is a retrospective observational cohort study that analyzed claims data from the Health Insurance Review and Assessment (HIRA) service in Korea between January 1, 2015 and December 31, 2019. Korea has a single payer national health system. The Korean National Health Insurance (NHI) covers approximately 97% of Koreans, while the 3% of remaining Koreans who cannot afford national insurance are covered by the Medical Aid Program since 1989, and HIRA has reviewed all claims data submitted by the NHI. The national health system manages and supervises the medical use information of all Koreans until death or loss of citizenship.

### Subjects

The study population consisted of lung cancer patients aged 18 years and older who were admitted to the ICU after receiving chemotherapy. We excluded patients who: (1) were >100 years of age, (2) have not received chemotherapy prior to admission to the ICU, and (3) presented with multiple primary lung cancer. We identified patients having cancer (code C34.x of the International Classification of Diseases 10^th^ revision) between January 1, 2015 and December 31, 2019. Follow-up was until December 05, 2020. We identified ICU admissions with codes of ICU services (codes AJ001–AJ590900). Chemotherapy was defined by Korean drug and anatomical therapeutic chemical codes (L01). Chemotherapy-related ICU admission was defined as receiving chemotherapy within 30 days before admission to the ICU.

### Ethics

The research protocol was approved by HIRA. Ethical approval for this study was exempted by the Dongguk University Hospital Institutional Review Board (DUIH 2020-10-040) because the authors only accessed previously collected data.

### Comorbidities and Concomitant Medical Therapy

Comorbidities were defined if claims data existed from 6 months before the index lung cancer diagnosis. Comorbidity diagnoses were defined using ICD-10 codes. The patients' underlying medical conditions were assessed using the Charlson comorbidity index (12). Concomitant medical therapy was defined by the procedure code of the Korean NHI.

### Statistical Analysis

The primary end point was all-cause ICU mortality. The secondary end points were 28-day, 60-day, and 90-day mortality. ICU admissions related to chemotherapy were divided into two groups depending on the type of chemotherapy: cytotoxic agents and targeted agents. Baseline variables and patient characteristics for each group are presented as percentages or as means with standard deviations. Between-group comparisons were estimated using χ^2^ tests for categorical data and Student's *t*-tests for continuous data. We used the Kaplan–Meier curve and log-rank test to compare survival data. We performed multivariate analysis using Cox regression adjusted for sex, age, Charlson comorbidity index, lines of treatment for cancer, ventilator support and renal replacement therapy, length from diagnosis with lung cancer and admission into the ICU, receiving surgery or radiation therapy prior to ICU admission, and the year of diagnosis with lung cancer to assess the effect of several factors on survival time. *P*-values of <0.05 were considered statistically significant. All analyses were carried out using R v.3.4.4 (using the packages “survival” and “ggplot2”).

## Results

From 2015 to 2019, 6,930 lung cancer patients received chemotherapy within 30 days before admission to the ICU. Most of the patients (85.4%, *n* = 5,919) received cytotoxic chemotherapy, and 14.5% (*n* = 1,011) received molecular targeted therapy (EGFR, 13.0% or ALK; tyrosine kinase inhibitor, 1.5%) (Table 1). Critically ill patients who received targeted therapy before ICU admission were older (66.7 vs. 65.1 years, *p* < 0.01), showed a female predominancy (56.9 vs. 19.6%, *p* < 0.01), and had fewer comorbidities (1.8 vs. 2.1, *p* < 0.01) than patients receiving cytotoxic chemotherapy. There were more patients with metastatic lung cancer in the targeted therapy group than in the cytotoxic group (75.5 vs. 66.0%, *p* < 0.01).

**Table 1:** Population characteristics according to chemotherapy.

**Variable**	**Total**	**Cytotoxic chemotherapy**	**Targeted chemotherapy**	***P*-value**
No. of patients	(*N* = 6,930)	5,919 (85.4%)	1,011 (14.6%)	
Age at diagnosis for lung cancer	65.0 ± 9.9	64.8 ± 9.5	66.2 ± 11.8	<0.01
Age at ICU admission	65.4 ± 9.8	65.1 ± 9.5	66.7 ± 11.8	<0.01
No. of females	1,735 (25.0%)	1,160 (19.6%)	575 (56.9%)	<0.01
No. of chemo-regimens before ICU	1.5 ± 0.9	1.6 ± 1.0	1.1 ± 0.4	<0.01
Metastatic lung cancer	4,671 (67.4%)	3,908 (66.0%)	763 (75.5%)	<0.01
CCI at ICU admission	10.0 ± 3.6	9.9 ± 3.7	10.5 ± 3.4	<0.01
CCI before diagnosis of lung cancer	2.1 ± 2.3	2.1 ± 2.3	1.8 ± 2.4	<0.01
Mechanical ventilation	2,961 (42.7%)	2,568 (43.4%)	393 (38.9%)	<0.01
Renal replacement therapy	476 (6.9%)	413 (7.0%)	63 (6.2%)	0.42
Neutropenia	35 (0.5%)	35 (0.6%)	0 (0%)	0.03
ICU LOS (days)	23.0 ± 51.0	22.3 ± 47.2	27.5 ± 69.3	0.02

*CCI, Charlson Comorbidity Index; ICU, intensive care unit; LOS, length of stay*.

Patients with targeted therapy were treated less frequently with other treatments for cancer, such as surgery (6.6 vs. 22.4%, *p* < 0.01) or radiation therapy (28.8 vs. 43.4%, *p* < 0.01) than those who received cytotoxic chemotherapy. Among patients who received targeted chemotherapy before admission to the ICU, 12.1% of patients had a history of cytotoxic chemotherapy. In comparison, 12.6% of patients who received cytotoxic chemotherapy just before ICU admission had a history of targeted therapy.

Critically ill patients who had received targeted therapy before admission to the ICU were treated less often with a mechanical ventilator (38.9 vs. 43.4%, *p* < 0.01). However, renal replacement therapy did not show a statistical difference in the two groups. In addition, patients who had received targeted therapy before ICU admission stayed significantly longer in the ICU than those who received cytotoxic chemotherapy (27.5 ± 69.3 vs. 22.3 ± 47.2, *p* = 0.02). Among survivors, the targeted therapy group also stayed longer in the ICU (26.0 ± 62.2) than those treated with cytotoxic therapy (21.2 ± 44.8, *p* = 0.05).

**Figure 1:**
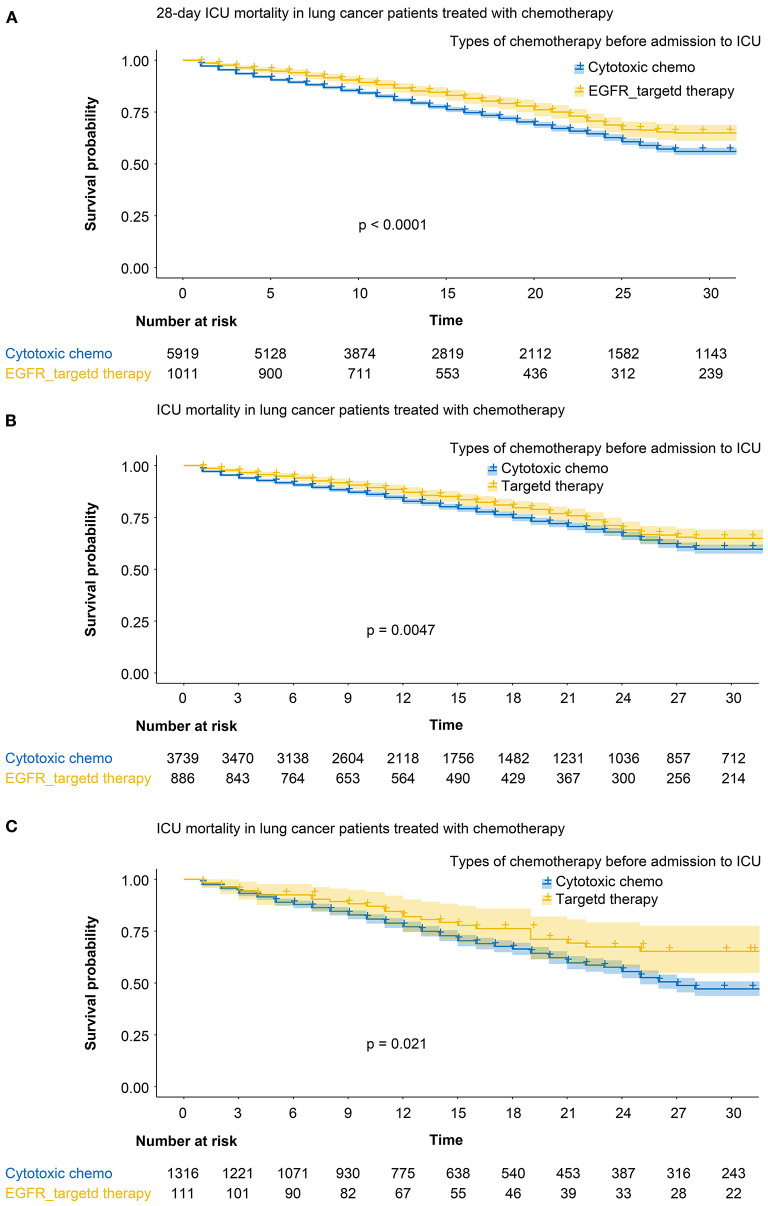
**(A)** 28-day mortality rate among the patients who received targeted agents (yellow line) and those who received cytotoxic chemotherapy (blue line). **(B)** Cumulative mortality rates in patients who received chemotherapy as a first-line regimen. Yellow line: targeted agents, blue line: cytotoxic chemotherapy. **(C)** Cumulative mortality rates in patients who received chemotherapy as a second-line regimen. Yellow line: targeted agents, blue line: cytotoxic chemotherapy.

The overall ICU mortality rate at 28, 60, and 90 days was significantly lower in patients who received targeted agents before ICU than those who received cytotoxic chemotherapy (Table 2). The 28-day mortality rate was 23.4% (237/1,011 patients) for patients who received targeted agents before admission to the ICU (log-rank test, *p* < 0.001; HR 0.72; 95% CI, 0.63–0.83, Figure 1A). Cumulative mortality rates at 28 days in patients who received chemotherapy as a first-line regimen (log-rank test, *p* < 0.001; HR for targeted chemotherapy 0.81; 95% CI, 0.72–0.92, Figure 1B) and in patients who received chemotherapy as a second-line regimen (log-rank test, *p* < 0.001; HR 0.60; 95% CI, 0.42–0.86, Figure 1C) were also statistically significant in both groups.

**Table 2:** ICU outcomes in critically ill patients with lung cancer.

**Variable**	**Total**	**Cytotoxic chemotherapy**	**Targeted chemotherapy**	***P*-value**
Overall ICU mortality	4,676	4,020 (67.9%)	656 (64.9%)	<0.01
Overall ICU mortality at 28-day	1,988	1,751 (29.6%)	237 (23.4%)	<0.01
Overall ICU mortality at 60-day	2,490	2,186 936.9%)	304 (30.1%)	<0.01
Overall ICU mortality at 90-day	2,560	2,246 (37.9%)	314 (31.1%)	<0.01

*ICU, intensive care unit*.

In the multivariable analysis, receiving targeted agents just before admission to the ICU (HR, 0.79; 95% CI, 0.67–0.90) was significantly associated with ICU mortality at 28 days. Age at ICU admission (HR, 1.03; 95% CI, 1.02–1.03) and being male (HR, 1.45; 95% CI, 1.29–1.63) were also significantly associated with ICU mortality at 28 days (Table 3). Respiratory failure requiring a mechanical ventilator in the ICU (HR, 3.30; 95% CI, 2.99–3.63) and renal failure (HR, 1.57; 95% CI, 1.36–1.80) requiring renal replacement therapy were independently associated with aggravated mortality.

**Table 3:** Cox proportional hazard analysis for ICU mortality.

	**Unadjusted HR**			**Adjusted HR**		
	**HR**	**95% CI**	***p*-value**	**HR**	**95% CI**	***p*-value**
Targeted therapy	0.72	0.64–0.81	<0.01	0.78	0.67–0.90	<0.01
Age at ICU admission	1.00	1.00–1.00	<0.01	1.03	1.02–1.03	<0.01
Male	1.4	1.30–1.60	<0.01	1.45	1.29–1.63	<0.01
No. of chemo-regimens before ICU	1.1	1.1–1.2	<0.01	0.86	0.83–0.92	<0.01
Years of diagnosis for lung cancer	0.87	0.84–0.89	<0.01	0.97	0.99–1.06	0.15
Metastatic lung cancer	0.82	0.75–0.89	<0.01	2.19	1.86–2.57	<0.01
CCI	0.95	0.94–0.96	<0.01	0.87	0.85–0.89	<0.01
Mechanical ventilation	2.00	1.80–2.10	<0.01	3.30	2.99–3.63	<0.01
Renal replacement therapy	1.60	1.40–1.80	<0.01	1.57	1.36–1.80	<0.01

*CCI, Charlson Comorbidity Index; CI, confidence interval; HR, hazard ratio; ICU, intensive care unit; LOS, length of stay*.

## Discussion

In this study, we investigated the effect of targeted therapies on outcomes of ICU treatments compared to cytotoxic chemotherapy in patients with lung cancer. Lung cancer patients receving targeted therapies had a significantly lower ICU mortality rate (HR, 0.79; 95% CI, 0.67–0.90 at 28 days), despite that they were significantly older and had a greater need for mechanical ventilation or renal replacement therapy in the ICU. This result suggests that the advent of targeted therapies could contribute to the improvement of treatment outcomes for critically ill patients with lung cancer.

Despite recommendations for limited therapeutic efforts in the management of critically ill patients with metastatic cancer admitted to the ICU, cancer patients account for about 20% of all patients admitted (13). Critically ill patients with lung cancer receving cytotoxic chemotherapy or targeted therapy may require ICU level of care, because most of the patients can have an advanced stage and aggressive cares may be a part of only life-sustaining treatments regarding as high mortality rates and side effects after ICU discharge (8). Recently, cancer patients receiving targeted therapy tend to continue treatment almost until death (14). This might be because targeted agents are more tolerable and more effective in advanced lung cancer than cytotoxic agents. Previous studies on critically ill patients with advanced lung cancer did not reflect recent trends in the emergence of targeted therapies (15). The advent of targeted therapies for lung cancer provides the impetus to reconsider the triage strategy and use of the ICU for cancer patients.

A recent study reported a significant improvement in the ICU mortality rates of cancer patients (16). The 28-day mortality in critically ill patients with cancer was reported to be about 50–60% in studies (17) up to the 2000's, but it was reported to be about 30–40% in a more recent study (18). Ostermann et al. (19) reported that the mortality of cancer patients improved from 31.3% in 2003–2005 to 26.0% in 2012–2014, while the change in mortality of non-cancer patients was insignificant, from 20.9 to 23.9%. The improvement in lung cancer mortality rate seems more likely to be a synergistic effect of cancer-related therapies rather than the overall improvement of diagnosis and treatment in the ICU.

Chen et al. reported that anticancer therapy in the ICU improved short-term ICU mortality for treatment-naïve patients with locally advanced lung cancer (20). However, in their subgroup analysis, they did not show a signifiant difference in ICU mortality at 28 days among those treated with cytotoxic chemotherapy and targeted therapy, but the number of study subjects was small. Our study shows that an overall survival benefit from ICU treatments could be achieved in patients receiving targeted therapy compared with lung cancer patients receiving cytotoxic chemotherapy. Consistent with previous studies, the presence of organ failure including respiratory failure or renal failure in critically ill patients was an important factor associated with mortality in critically ill patients with lung cancer (21). These results suggest that lung cancer patients receiving targeted therapy could be considered more suitable for ICU treatment.

This study had several limitations. First, we analyzed administrative data. Therefore, it was not available about sociodemographic characteristics and questionnaire data and not seletable for genetic and behavioral disorders. In addition, information on cancer staging or subtypes, such as SCLC or NSCLC, was also not detailed. Based on whether the lung cancer had metastasized, we could assume whether the cancer was at a more advanced stage. However, this cannot replace direct staging of lung cancer. Second, in this study, most patients with SCLC would have been classified into the cytotoxic chemotherapy group. Third, we had no information on the reasons for critical care. Therefore, our results should be interpreted with caution.

Still, this study might be meaningful because it is the first study to show that patients who received targeted cancer therapy could have better outcomes in the ICU, unlike those treated with drugs such as cytotoxic chemotherapy. However, further validation is needed to confirm our results. The effect of cancer stage, which was not identified in this study, on improving ICU treatment outcomes of targeted therapy also needs to be confirmed in future studies.

## Conclusion

It remains difficult to predict which cancer patients will recover with critical care. For continuous improvement of ICU treatment, it is necessary to consider the patient's current condition, including the type of chemotherapy, the patient's treatment intention, the responsiveness to cancer treatment, the future treatment plan, the level of ICU treatment, the possibility of lung treatment, and other factors. Future studies would shed more light on the underlying interactions of these parameters and could help in developing novel diagnostic and therapeutic strategies for lung cancer management.

## Data Availability Statement

The datasets presented in this article are not readily available because our database was provided by HIRA. We only accessed deidentified previously collected administrative data. Requests to access the datasets should be directed to https://opendata.hira.or.kr.

## Ethics Statement

The studies involving human participants were reviewed and approved by Dongguk University Hospital Institutional Review Board (DUIH 2020-10-040). The Ethics Committee waived the requirement of written informed consent for participation. Written informed consent was not obtained from the individual(s) for the publication of any potentially identifiable images or data included in this article.

## Author Contributions

All authors listed contributed to the conception and design of the work, to the acquisition, analysis, interpretation of the data, to drafting the manuscript and approved it for publication.

## Funding

This work was supported by the Dongguk University Research Program of 2021 and the National Research Foundation of Korea grant funded by Korea government (NRF-2021R1I1A3056129 and NRF-2020R1C1C1009091).

## Conflict of Interest

The authors declare that the research was conducted in the absence of any commercial or financial relationships that could be construed as a potential conflict of interest.

## Publisher's Note

All claims expressed in this article are solely those of the authors and do not necessarily represent those of their affiliated organizations, or those of the publisher, the editors and the reviewers. Any product that may be evaluated in this article, or claim that may be made by its manufacturer, is not guaranteed or endorsed by the publisher.
